# TGFβ signalling maintains Sox4+ astrocyte progenitors and restricts the early differentiation of BLBP+ astrocytes in the mouse dentate gyrus

**DOI:** 10.3389/fncel.2026.1882016

**Published:** 2026-07-01

**Authors:** Kyoji Ohyama, Shoichiro Omura, Natsumi Takayama, Tokiharu Takahashi

**Affiliations:** Department of Histology and Neuroanatomy, Tokyo Medical University, Tokyo, Japan

**Keywords:** astrocyte progenitor, p57, BLBP/Fabp7, dentate gyrus, differentiation, Olig2, Sox4, TGFb (transforming growth factor-beta)

## Abstract

Astrocytes in the mouse hippocampal dentate gyrus (DG) derive, at least in part, from BLBP+ cells, but the mechanism that controls the onset of their differentiation remains poorly understood. To address this, we first characterised the developmental dynamics of BLBP+ cells using Ki67 and the cyclin-dependent kinase inhibitor p57. Ki67+/BLBP+ proliferative progenitors peaked at P1 and declined by P3–P6, when p57+/BLBP+ early-differentiating astrocytes instead accumulated. This temporal switch prompted us to examine what maintains the proliferative pool and restricts premature differentiation. We identified Sox4, a downstream transcription factor of TGFβ signalling, as a selective marker of the undifferentiated astrocytic lineage in the developing DG. At E15–E17, Sox4 was co-expressed with BLBP and pVimentin in basal radial glia (bRG), and at P1–P6 with BLBP, Sox3 and Ki67 in proliferative glial progenitors including the Olig2+/BLBP+ astrocyte progenitors (ASPs) we recently identified. Our data further suggest that Sox4 marks ASPs but not either NeuroD+ neuronal progenitors or p57+/BLBP+ early-differentiating astrocytes. From P14 onwards, Sox4 expression persisted in a subpopulation of BLBP+ radial glia-like cells (RGLs) at the SGZ and in BLBP+ cells in the molecular layer. Together, these data suggest that Sox4 selectively marks BLBP+ glial progenitors (bRG, RGLs and ASPs) but not their differentiated progeny. Consistent with an autocrine TGFβ1-Sox4 pathway described in glioma-initiating cells, TGFβ1 was co-expressed with BLBP in bRG, RGLs and ASPs. Blockade of TGFβ signalling with SB431542 in DG explants depleted Sox4+ progenitors and, in turn, promoted the accumulation of p57+/BLBP+ astrocytes. Our data thus suggest that TGFβ signalling maintains Sox4+ progenitors and thereby restricts the early differentiation of BLBP+ astrocytes in the mouse DG.

## Introduction

For the last decades, it has been assumed that GFAP+ cells represent entire population of astrocytes, and that increased expression of GFAP in pathological conditions marks reactive astrocytes that respond to injury-induced neuroinflammation. However, recent advances in single-cell transcriptomics (scRNAseq) have built up “cell atlases” in various organs including the central nervous system (CNS) and changed our view of cellular heterogeneity. Astrocytes are no exception. Growing bodies of evidence showed that astrocytes comprise a heterogeneous population (Herrero-Navarro et al., 2021). For instance, GFAP−/crym+/S100β+ striatal astrocytes have been shown to gate perseverative behaviour (Ollivier et al., 2024). The oligodendrocyte progenitor marker Olig2 is expressed in astrocytes that are mostly GFAP- cells in both developing and adult mouse brain (Marshall et al., 2005; Tatsumi et al., 2018; Omura et al., 2024), and Olig2+ astrocytes migrate along blood vessels via ECM-integrin signalling (Tabata et al., 2022). In the embryonic mouse dentate gyrus (DG), BLBP/Fabp7, originally identified as a radial glial marker (Feng et al., 1994), is expressed mostly in the fimbria and subpial region of the DG. In contrast, soon after birth, one-third of the *gfap*-GFP+ radial glia-like cells (RGLs) start expressing BLBP (Matsue et al., 2018).

Similarly, we recently identified BLBP+/Olig2+ astrocyte progenitors (ASPs) that are, to a lesser extent, *gfap*-GFP+ and mostly GFAP−. These BLBP+ cells contribute to astrocytes in the late embryonic and early postnatal DG, though their number substantially decreases by postnatal day 14 (P14) (Omura et al., 2024). Despite these advances, however, it remains poorly understood how the differentiation of BLBP+ astrocytes is controlled.

Among the signals that maintain neural stem/progenitor pools, TGFβ has emerged as a key regulator. TGFβ ligands bind to tetrameric receptor complexes composed of TGFβ type I and type II receptors, signaling through various downstream effectors such as pSmad2/3 and non-canonical pERK (Deng et al., 2024). We previously showed that pSmad3, a downstream effector of TGFβ signalling, is expressed in a subpopulation of *gfap*-GFP+ bRG, RGLs and astrocytes in the developing DG (Ohyama et al., 2023). In glioma-initiating cells, autocrine TGFβ signalling maintains tumorigenicity through the induction of Sry-related HMG-box (Sox) transcription factors, in particular Sox4 (Ikushima et al., 2009). Sox4 in turn acts as a master regulator of epithelial–mesenchymal transition (EMT) by controlling Ezh2-mediated epigenetic reprogramming (Tiwari et al., 2013), and pSmad3 and Sox4 can cooperatively regulate shared target genes. These findings raise the possibility that a TGFβ–Sox4 axis operates in BLBP+ glial progenitors of the developing DG to maintain their proliferative, undifferentiated state. Whether such a mechanism controls the onset of BLBP+ astrocyte differentiation, however, has not been addressed.

In this study, we first characterised the developmental dynamics of BLBP+ cells in the mouse DG using Ki67 and the cyclin-dependent kinase inhibitor p57, and identified a temporal switch from Ki67+/BLBP+ proliferative progenitors at P1 to p57+/BLBP+ early-differentiating astrocytes at P3–P6. To explore what maintains the proliferative pool and restricts premature differentiation, we examined Sox4 expression and found that it selectively marks BLBP+/pVimentin+ bRG, BLBP+ RGLs, and Olig2+/BLBP+ ASPs, but is absent from p57+/BLBP+ differentiating astrocytes and from NeuroD+ neuronal progenitors. TGFβ1 was co-expressed with BLBP in these Sox4+ progenitors, and pharmacological blockade of TGFβ signalling in DG explants depleted Sox4+ progenitors and promoted the accumulation of p57+/BLBP+ astrocytes. Our data thus indicate that TGFβ1 signalling maintains Sox4+BLBP+ glial progenitors and restricts the early differentiation of BLBP+ astrocytes in the developing mouse DG.

## Materials and methods

### Animals

C57BL6/Ncrl and *gfap*-GFP mice that express GFP under the control of the mouse gfap promoter (Suzuki et al., 2003) were housed under standard conditions (12 h light/dark cycle) at the animal care facility of Tokyo Medical University. All experiments were conducted in accordance with the guidelines of the Institutional Animal Care and Use Committees and conformed to the National Institutes of Health Guide for the Care and Use of Laboratory Animals (NIH Publication No. 80-23) revised in 1996. Every effort was made to minimize the number of animals used and their suffering. Embryos and pups from C57BL6/NCrl and the above *gfap*-GFP transgenic mice were used. The day on which a vaginal plug was found was designated as embryonic day 0.5 (E0.5) and the day of birth was designated as postnatal day 0.5 (P0.5).

### Tissue preparation

Embryos were harvested at E14.5–E18.5 from pregnant mice, and early postnatal day 1–21 (P1–21) mice were anesthetized and sacrificed following approved ethical guidelines as described above. Brain tissues were then isolated and fixed with 4% paraformaldehyde (PFA) in 0.1 M phosphate buffer (PB), pH 7.4, by immersion. P14 and P21 mice were transcardially perfused with 15 mL of PBS, followed by 15–30 mL of 4% PFA in 0.1 M PB, pH 7.4, at room temperature for 5–10 min. Brain tissues were isolated and post-fixed by immersion in 4% PFA. After fixation, brains were washed with PBS and immersed in 30% sucrose/0.1 M PB. Forebrains were embedded in OCT compound and stored at −70 °C. Cryosections were cut at a thickness of 25 μm.

### Antibodies

The antibodies used in this study are as follows: guinea pig anti-BLBP (Frontier institute, MSFR100290, 1:1,000); chick anti-GFP (Abcam, ab13970, 1:5,000); rabbit anti-Ki67 (Novocastra, NCL-Ki67p, 1:1,000); goat anti-NeuroD (N-19) (Santa Cruz, sc-1084 Santa Cruz, sc-1084, 1:1,000); rabbit anti-Olig2 (Millipore, AB9610, 1:1,000); goat anti-Olig2 (R&D systems, AF2418, 1:1,000); rabbit anti-p57 (Sigma, P0357, 1:250); rabbit anti-pSmad3 (Millipore, 07-1389, 1:1,000); mouse anti- phosphorylated vimentin (pVim) (MBL, D076-3S, 1:250); goat anti-Sox2 (R&D systems, AF2018, 1:1,000); rabbit anti-Sox3 serum (gift from T. Edlund, 1:1,000); rabbit anti-Sox4 (Millipore, AB5803, 1:500–1,000); goat anti-Sox9 (R&D systems, AF3075, 1:1,000); rabbit anti-TGFβ1 (Santa cruz, sc-146, 1:100). Species-appropriate Alexa Fluor-conjugated secondary antibodies (Invitrogen) were used as in the followings. Alexa fluor 555 donkey anti-rabbit IgG (Jackson ImmunoResearch, 711-565-152, 1:1,000); Alexa fluor 488 donkey anti-mouse IgG (Jackson ImmunoResearch, 715-545-151, 1:1,000); Alexa fluor 488 donkey anti-goat IgG (Jackson ImmunoResearch, 705-545-147, 1:1,000); Alexa fluor 488 donkey anti-guinea pig IgG (Jackson ImmunoResearch, 706-545-148, 1:1,000); Alexa fluor 647 donkey anti-goat IgG (Jackson ImmunoResearch, 705-605-147, 1:1,000); Alexa fluor 647 donkey anti-guinea pig IgG (Jackson ImmunoResearch, 706-605-148, 1:1,000).

### Immunofluorescent labelling

Cryosections were processed for immunohistochemistry as previously described (Ohyama et al., 2023). Briefly, antigen retrieval was carried out with Histo VT One (Nacalai, Japan) following manufacturer’s instructions. Cryosection of the hippocampus were incubated with primary antibodies overnight at 4 °C. After three washes with PBS, the sections were incubated with secondary antibodies with DAPI for 45 min at room temperature. After three washes with PBS, the sections were mounted with Vectashield (Vector lab, CA). Images were taken with a Zeiss LSM900 confocal microscope. In some cases, fluorescence images were digitally zoomed at 0.5x to 2x. Stacks of optical sections (1.5 μm thickness/optical section) were obtained at 1.0 μm increments on the z-axis using an 20x objective. Images were corrected for brightness and contrast and composed using Zeiss Image Browser, ZEN software (Zeiss, Thornwood, NY) and Adobe Photoshop 2025 (San Jose, CA). Mice (*n* = 3–5) were examined for individual experiments and, for quantification of some experiments, 4–19 sections were analysed for each using Fiji from image J as described previously (Ohyama et al., 2023; Ohyama et al., 2024). Mean ± SE is given in the results.

### Explant culture

Mouse hippocampi were dissected out at postnatal day 0.5 (designated as P0) and cut into 4 pieces. Each hippocampus was embedded in collagen gel and cultured with 10 μM SB431542 (Tocris Bioscience, 1614) or DMSO for either 6 days or 12 days in the mixture of DMEM/F12 with Mito serum (BD, New Jersey, United States) and Glutamax (GIBCO), at 37 °C in 5% CO_2_, as described previously (Place et al., 2022). Cultured explants were then processed for immunofluorescent labelling.

### Statistical analysis

Quantitative data are presented as mean ± SE. Comparisons between two groups (DMSO versus SB431542) were performed using two-tailed Student’s *t*-tests in GraphPad Prism 11 (Dotmatics). *p* values < 0.05 were considered statistically significant. Sample sizes (*n*) and the number of sections analysed are reported in the figure legends and Results.

## Results

### BLBP+ cells in the developing mouse DG undergo a temporal switch from a Ki67+ proliferative state to a p57+ early-differentiated state

BLBP+ cells contribute to astrocytes in the developing DG (Omura et al., 2024), but when and how these cells transition from proliferation to differentiation has not been characterised. To address this, we examined the developmental dynamics of BLBP+ cells using the proliferation marker Ki67 and the cyclin-dependent kinase inhibitor p57, which marks cells that have exited the cell cycle and begun differentiation.

**Figure 1:**
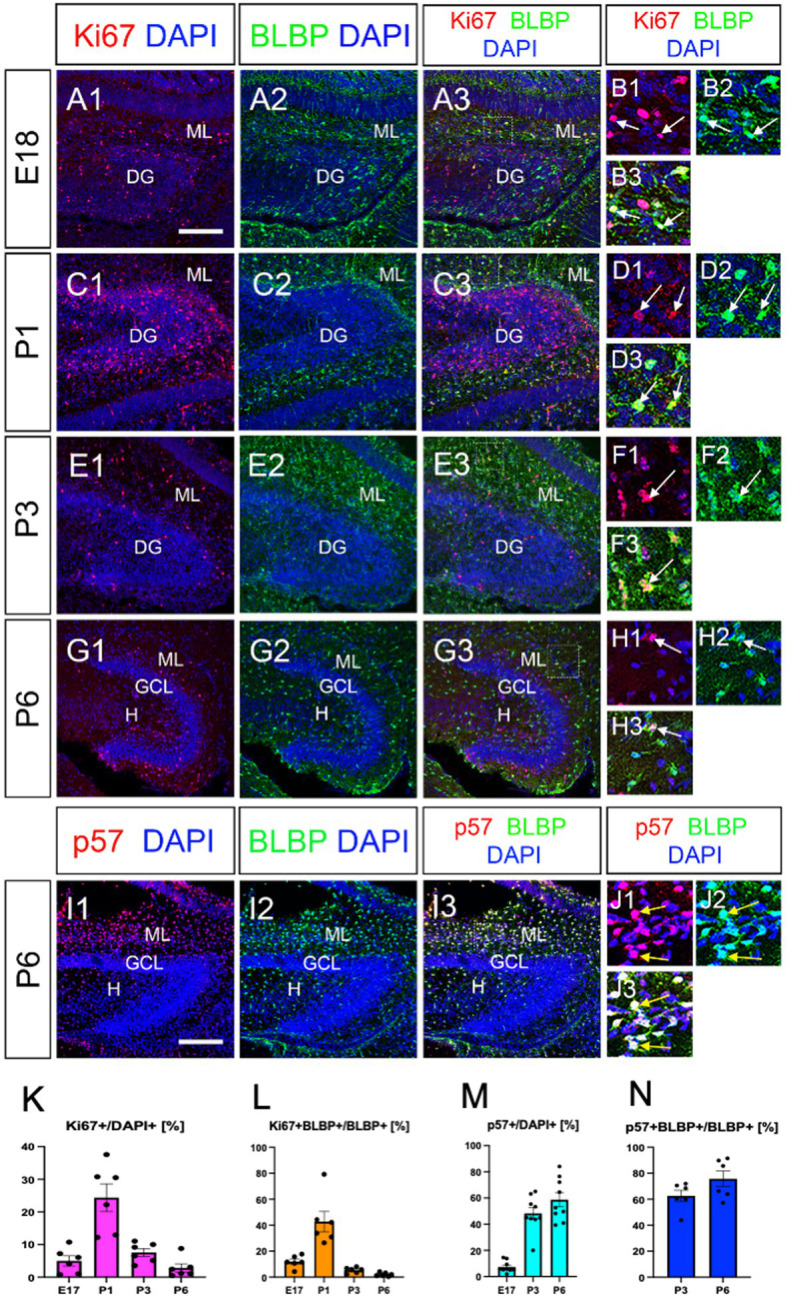
Temporal switch from Ki67+ proliferative state to p57 + early-differentiating state in BLBP+ cells of the developing DG. **(A1–A3,B1–B3)** Ki67 (red) and BLBP (green) immunolabelling at E18. **(C1–C3,D1–D3)** The same labelling at P1. **(E1–E3,F1–F3)** The same labelling at P3. **(G1–G3,H1–H3)** The same labelling at P6. **(I1–I3 J1–J3)** p57 (red) and BLBP (green) immunolabelling at P6. Boxed area in **A3**, **C3**, **E3**, **G3** and **I3** are shown at higher magnification in **B1–B3**, **D1–D3**, **F1–F3**, **H1–H3**, and **J1–J3**, respectively. **(K)** Quantification of Ki67+ cells as a percentage of DAPI+ double-positive cells at E17, P1, P3 and P6 (*n* = 3 mice; 6 sections per stage) **(L)** Quantification of Ki67+ BLBP+ double-positive cells as a percentage of BLBP+ cells (*n* = 3 mice; 6 sections per stage). **(M)** Quantification of p57+ cells as a percentage of DAPI+ cells at E17, P3 and P6 (*n* = 4 mice; 9 sections per stage). **(N)** Quantification of p57+/BLBP+ double-positive cells as a percentage of BLBP+ cells at P3–P6 (*n* = 4 mice; 6 sections per stage). DG, dentate gyrus; ML, molecular layer; GCL, granule cell layer; H, hilus. Scale bars: 200 μm in A1 (applies to **A1–A3**, **C1–C3**, **E1–E3**, **G1–G3**) and I1 (applies to **I1–I3**).

BLBP+ cells were sparse at E17, increased between E18 and P3, declined from P6 onwards (Figures 1A–H). Total Ki67+ proliferative cells peaked at P1 (24.3% ± 4.2% of DAPI+ cells, *n* = 3) and fell sharply by P3 (7.5% ± 1.2%) and P6 (2.8% ± 1.2%) (Figure 1K). A similar profile was observed for Ki67+/BLBP+ double-positive cells, which peaked at P1 (42.8% ± 7.7% of BLBP+ cells) and decreased by P3 (5.3% ± 0.8%) and P6 (2.1% ± 0.5%) (Figure 1L). In contrast, p57 + cells were rare at E17 (4.8% ± 0.6%) but accumulated markedly by P3 (42.6% ± 12.5%) and P6 (54.7% ± 9.5%) (Figures 1I,J,M). The majority of BLBP+ cells at P3 and P6 were p57 + (62.5% ± 4.2% at P3; 75.7% ± 6.0% at P6) (Figures 1I,J,N), indicating that most BLBP+ cells present during this window had exited the cell cycle and entered early differentiation.

Together, these data demonstrate a temporal switch in the BLBP+ lineage: Ki67+/BLBP+ proliferative progenitors predominate at P1, whereas p57+/BLBP+ early-differentiating astrocytes accumulate at P3–P6. This switch raised the question of what maintains the proliferative pool and restricts its premature differentiation, prompting us to search for transcription factors selectively expressed in the proliferative BLBP+ population.

### Sox4 is co-expressed with BLBP and pVimentin in basal radial glia of the embryonic DG

To identify transcription factors that selectively mark the proliferative BLBP+ population, we turned to Sox4, a downstream target of TGFβ signalling that maintains the stemness of glioma-initiating cells (Ikushima et al., 2009) and cooperates with pSmad3 in regulating shared target genes.

**Figure 2:**
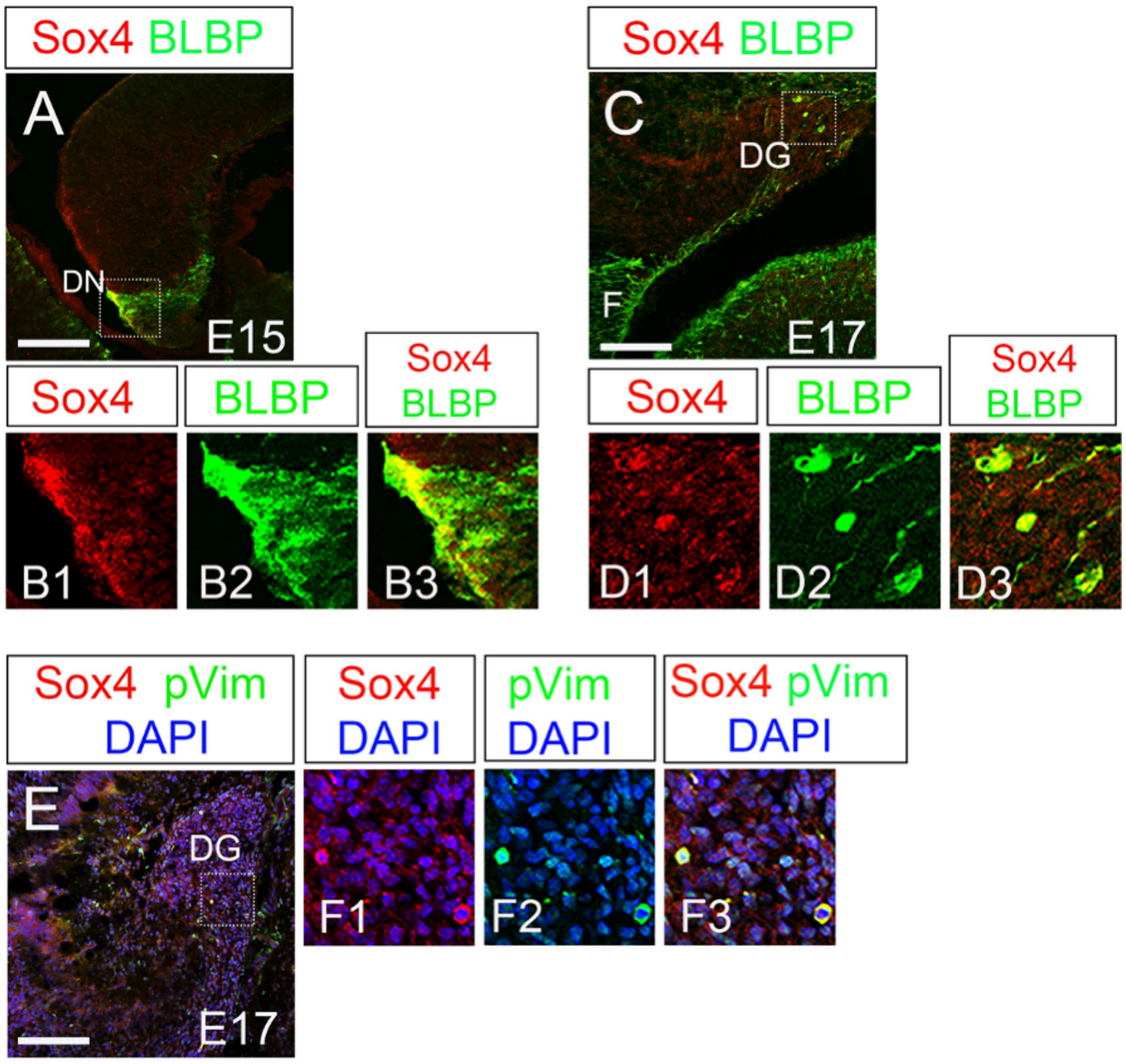
Sox4 is co-expressed with BLBP and pVimentin in basal radial glia of the embryonic DG. **(A,B1–B3)** Sox4 (red) and BLBP (green) immunolabelling at E15. Sox4 is expressed around the dentate notch (DN) and co-localises with BLBP. **(C,D1–D3)** Sox4 (red) and BLBP (green) immunolabellingat E17 in the forming DG, showing nuclear Sox4 signal in BLBP+ cells. **(E,F1–F3)** Sox4 (red) and phosphorylated vimentin (pVim, green) immunolabelling at E17, with DAPI counterstain (blue). Sox4+/pVim+ cells are observed in mitosis. Boxed area in **A**, **C** and **E** are shown at higher magnification in **B1–B3**, **D1–D3**, and **F1–F3**, respectively. DG, dentate gyrus; DN, dentate notch; F, fimbria. Scale bars: 200 μm in **A**, **C**, **E**.

In the embryonic DG, Sox4 was expressed around the dentate notch (DN) at E15 and was co-localised with BLBP (Figures 2A,B1–B3). Given its cytoplasmic expression as descried previously (Lai et al., 2011), the data suggest that Sox4-mediated transcriptional regulation is not activated at this stage. At E17, nuclear Sox4 signal was clearly detected in BLBP+ cells in the forming DG (Figures 2C,D1–D3). To test whether these Sox4+/BLBP+ cells correspond to proliferative basal radial glia (bRG), we co-stained with phosphorylated vimentin (pVim), a marker of mitotic bRG (Ohyama et al., 2023). Sox4 was co-expressed with pVim at E17, and Sox4+/pVim+ cells were under cell captured in mitosis (Figures 2E,F1–F3), confirming that Sox4 is expressed in proliferating bRG during embryonic DG development.

We next asked whether pSmad3, another TGFβ effector we previously described in *gfap*-GFP+ bRG (Ohyama et al., 2023), is similarly expressed in the pVim+ bRG of the embryonic DG. At E16, pSmad3 was co-expressed with pVim in mitotic bRG (Figures 3A,B1–B3) and with BLBP in the same cell population (Figures 3C,D1–D3). Consistent with the onset of Sox4 expression in BLBP+ and pVim+ bRG during embryonic period (Figures 2A–F3), glial progenitor markers Sox2 and Sox3 are co-expressed in BLBP+ cells at E17 (Figures 3E,F1–F4). Further, at P1, pVim+ cells co-expressed both Sox2 and Sox3 in the molecular layer and hilus of the DG (Figures 3G,H1–H7), consistent with their identity as proliferative glial progenitors.

**Figure 3:**
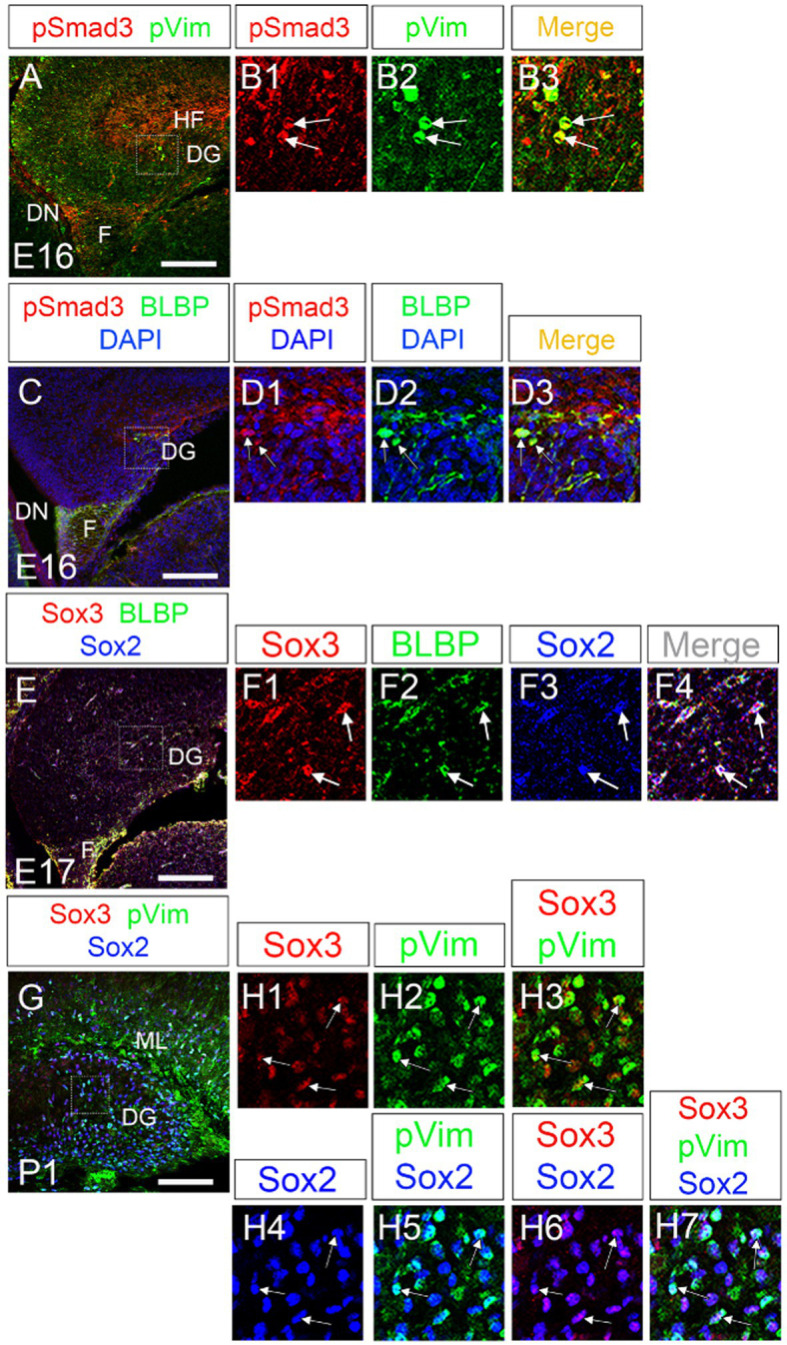
pSmad3 is expressed in pVim+/BLBP+ bRG, and pVim+ cells co-express Sox2 and Sox3 in the developing DG. **(A,B1–B3)** pSmad3 (red) and pVim (green) immunolabelling at E16. Arrows indicate pSmad3+/pVim+ mitotic bRG. **(C,D1–D3)** pSmad3 (red) and BLBP (green) immunolabelling with DAPI counterstain (blue) at E16. Arrows indicate pSmad3+/BLBP+ bRG. **(E,F1–F4)** Triple immunolabelling for Sox3 (red), BLBP (green) and Sox2 (blue) at E17. The arrows indicate Sox3+/BLBP+/Sox2+ cells around the DG. **(G,H1–H7)** Triple immunolabelling for Sox3 (red), pVim (green) and Sox2 (blue) at P1. Arrows indicate Sox3+/pVim+/Sox2+ cells in the hilus. Boxed areas in **A**, **C**, **E**, and **G** are shown at higher magnification in **B1–B3**, **D1–D3**, **F1–F4**, and **H1–H7**, respectively. DG, dentate gyrus; ML, molecular layer; HF, hippocampal fissure; DN, dentate notch; F, fimbria. Scale bars: 200 μm in **A**, **C**, **E**, **G**.

Together, these data show that Sox4 and pSmad3, two components of the TGFβ signalling pathway, are co-expressed with BLBP and pVim in proliferative bRG of the embryonic DG, raising the possibility that a TGFβ–Sox4 axis operates in this population.

### Sox4 selectively marks Olig2+/BLBP+ astrocyte progenitors and BLBP+ RGLs in the postnatal and adult DG

**Figure 4:**
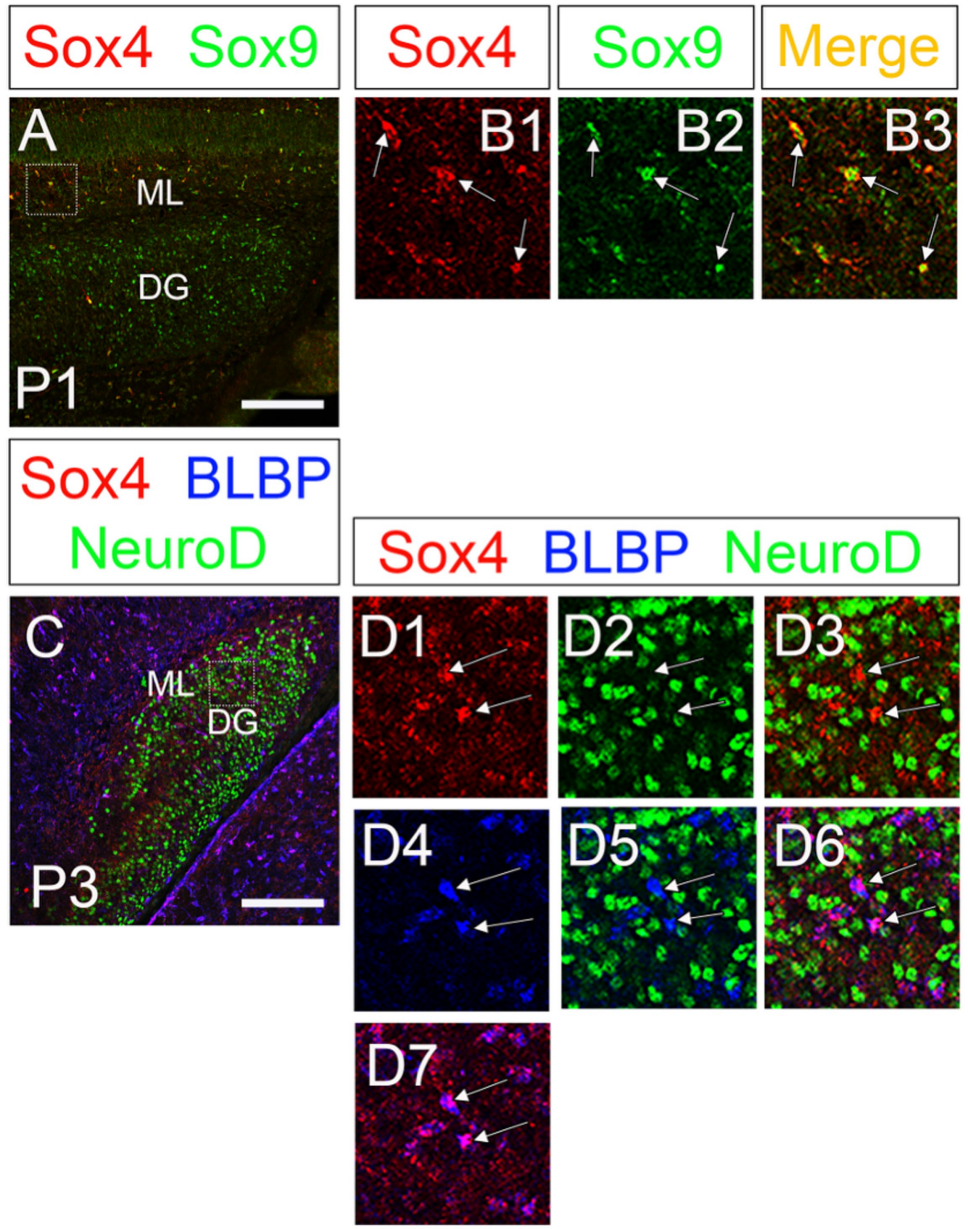
Sox4 is co-expressed with the astrocytic lineage marker Sox9 but not with the neuronal progenitor marke NeuroD in the postnatal DG. **(A,B1–B3)** Sox4 (red) and Sox9 (green) immunolabelling at P1. Arrows indicate Sox4+/Sox9+ double-positive cells. **(C,D1–D7)** Triple immunolabelling for Sox4 (red), BLBP (blue) and Neuro D (green) at P3. Sox4+/BLBP+ cells (arrows) do not co-express NeuroD. Boxed areas in **A** and **C** are shown at higher magnification in **B1–B3** and **D1–D7**, respectively. DG, dentate gyrus; ML, molecular layer. Scale bars: 200 μm in **A** and **C**.

We next examined whether Sox4 expression persists in the postnatal DG and whether it distinguishes astrocytic progenitors from other cell lineages. At P1, Sox4 was co-expressed with Sox9, a broad marker of the astrocytic lineage (Figures 4A,B1–B3). In contrast, Sox4+/BLBP+ cells did not co-express a neuronal progenitor marker NeuroD at P3 (Figures 4C,D1–D7), indicating that Sox4 expression is restricted to the astrocytic lineage and excluded from the neuronal lineage of the DG.

**Figure 5:**
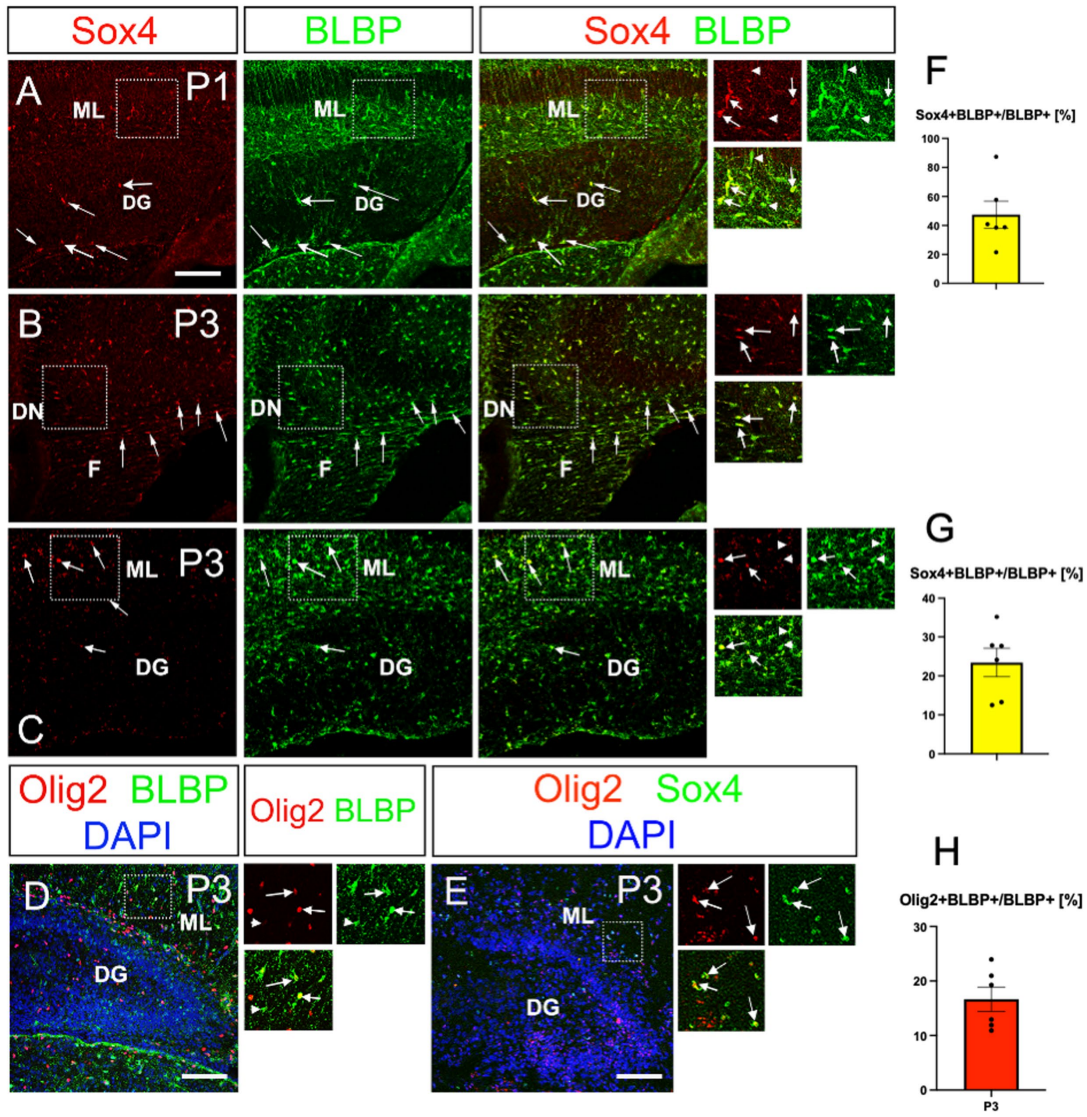
Sox4 marks Olig2+/BLBP+ astrocyte progenitors in the early postnatal DG. **(A)** Sox4 (red) and BLBP (green) immunolabelling at P1. Arrows indicate Sox4+/BLBP+ cells. The boxed area is shown at higher magnification on the right. **(B)** Sox4 (red) and BLBP (green) immunolabelling at P3, in the region of the dentate notch. **(C)** Sox4 (red) and BLBP (green) immunolabelling at P3, in the molecular layer of the DG. Arrows indicate Sox4+/BLBP+ cells; arrowheads indicate Sox4+/BLBP- cells. **(D)** Olig2 (red) and BLBP (green) immunolabelling with DAPI counterstain (blue) at P3. Arrows indicate Olig2+/BLBP+ cells; arrowheads indicate Olig2-/BLBP+ cells. **(E)** Olig2 (red) and Sox4 (green) immunolabelling with DAPI counterstain (blue) at P3. Arrows indicate Olig2+/Sox4+ cells. **(F,G)** Quantification of Sox4+/BLBP+ cells as a percentage of BLBP+ cells: 47.4% ± 9.2% at P1; 23.4% ± 3.6% at P3 (*n* = 3 mice; 6 sections per stage). **(H)** Quantification of Olig2+/BLBP+ cells as a percentage of BLBP+ cells at P3: 16.6% ± 2.2% (*n* = 3 mice; 6 sections). DG, dentate gyrus; ML, molecular layer; DN, dentate notch; F, fimbria. Scale bars: 200 μm in **A**, **B**, **C**, **D**, and **E**.

A substantial fraction of BLBP+ cells in the early postnatal DG were Sox4 + (47.4% ± 9.2% of BLBP+ cells at P1; 23.4% ± 3.6% at P3; Figures 5A–C,F,G). Given that Sox4 + cells expressed the astrocytic lineage markers BLBP and Sox9 (Figures 4A,B1–B3), we examined whether they correspond to the Olig2+/BLBP+/Sox9+ proliferative astrocyte progenitors (ASPs) we recently identified (Omura et al., 2024). A subset of BLBP+ cells co-expressed Olig2 at P3 (16.6% ± 2.2% of BLBP+ cells; Figures 5D,H), and Sox4 was indeed co-expressed with Olig2 in the same anatomical region (Figure 5E). Together, these data identify Sox4+/Olig2+/BLBP+ cells as ASPs in the postnatal DG, a population distinct from the p57+/BLBP+ early-differentiating astrocytes described in Figure 1.

**Figure 6:**
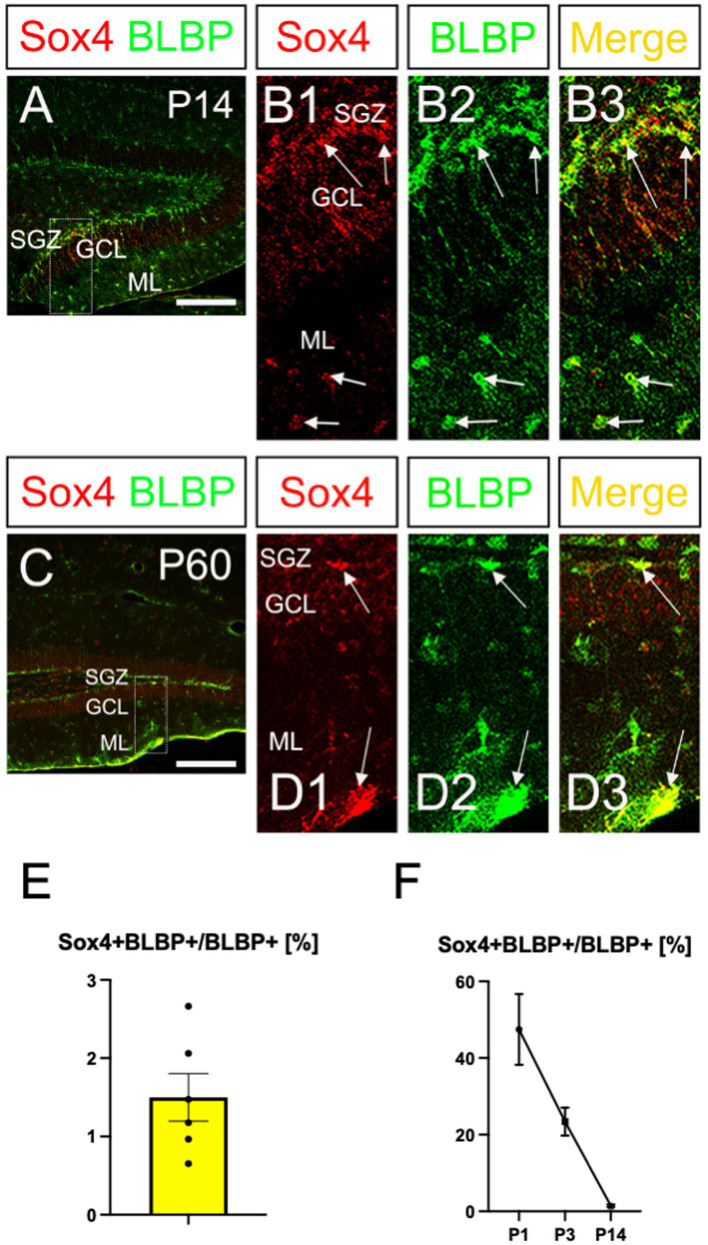
Sox4 expression persists in BLBP+ RGLs and astrocytes in the postnatal and adult DG. **(A,B1–B3)** Sox4 (red) and BLBP (green) immunolabelling at P14. Arrows indicate Sox4+/BLBP+ radial glia-like cells (RGLs) at the subgranular zone (SGZ) and astrocytes in the molecular layer (ML). **(C,D1–D3)** Sox4 (red) and BLBP (green) immunolabelling at P60. Arrows indicate Sox4+/BLBP+ RGLs at the SGZ and Sox4+/BLBP+ astrocytes in the molecular layer. Boxed areas in **A** and **C** are shown at higher magnification in **B1–B3** and **D1–D3**, respectively. **(E)** Quantification of Sox4+/BLBP+ cells as a percentage of BLBP+ cells: 1.4% ± 0.3% at P14 (*n* = 3 mice, 6 sections). **(F)** A decrease in the ratio of Sox4+BLBP+ cells to BLBP+ cells at P1, P3, and P14. DG, dentate gyrus; SGZ, subgranular zone; GCL, granule cell layer; ML, molecular layer. Scale bars: 200 μm in **A** and **C**.

Sox4 expression in the BLBP+ astrocytic lineage persisted beyond the early postnatal period. At P14, Sox4 was detected in BLBP+ RGLs at the SGZ and in BLBP+ astrocytes in the ML (1.4 ± 0.3% of BLBP+ cells; Figures 6A,B1–B3,E). This pattern was maintained in the adult DG at P60 (Figures 6C,D1–D3), indicating that Sox4 continues to mark the BLBP+ astrocytic lineage into adulthood. Furthermore, our findings demonstrate a significant postnatal decline in the population of Sox4 + BLBP+ astrocytic progenitors (Figure 6F).

Together with the embryonic data (Figure 2), these findings show that Sox4 selectively marks BLBP+ progenitors throughout the astrocytic lineage of the DG, from bRG in the embryo, through Olig2+/BLBP+ ASPs in early postnatal stages, to RGLs at the SGZ in the adult, while being excluded from p57+/BLBP+ differentiating astrocytes and NeuroD+ neuronal progenitors.

### TGFβ signalling maintains Sox4+ progenitors and restricts the early differentiation of BLBP+ astrocytes

**Figure 7:**
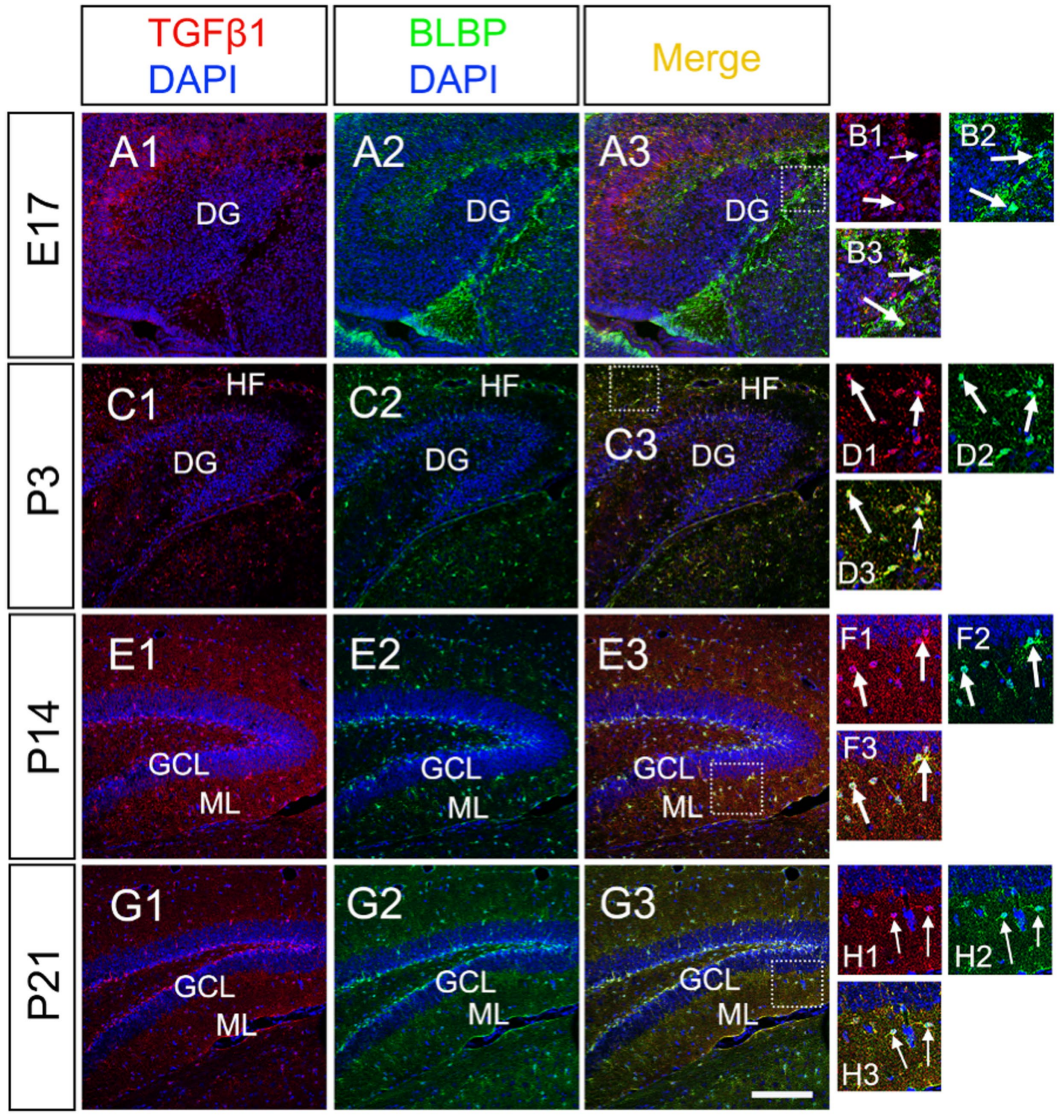
TGFβ1 is co-expressed with BLBP+ in the developing and adult DG. **(A1–A3,B1–B3)** TGFβ1 (red) and BLBP (green) immunolabelling with DAPI counterstain (blue) at E17. Arrows in **B1–B3** indicate TGFβ1+/BLBP+ cells. **(C1–C3,D1–D3)** The same labelling at P3. Arrows in **D1–D3** indicate TGFβ1+/BLBP+ cells around the forming DG. **(E1–E3,F1–F3)** The same labelling at P14. Arrows in **F1–F3** indicate TGFβ1+/BLBP+ astrocytes. **(G1–G3,H1–H3)** The same labelling at P21. Arrows in **H1–H3** indicate TGFβ1+/BLBP+ astrocytes. Boxed areas in **A3**, **C3**, **E3**, and **G3** are shown at higher magnification in **B1–B3**, **D1–D3**, **F1–F3**, and **H1–H3**, respectively. DG, dentate gyrus; HF, hippocampal fissure; GCL, granule cell layer; ML, molecular layer. Scale bar: 200 μm in **G3**.

Given the autocrine TGFβ-Sox4 signalling described in glioma-initiating cells (Ikushima et al., 2009), we examined whether TGFβ1 is expressed in the Sox4+/BLBP+ progenitor population of the DG. TGFβ1 was co-expressed with BLBP in bRG, at E17 (Figures 7A1–A3,B1–B3), in BLBP+ cells around the forming DG at P3 (Figures 7C1–C3,D1–D3), and in BLBP+ RGLs and astrocytes at P14 and P21 (Figures 7E1–E3,F1–F3,G1–G3,H1–H3). Thus, TGFβ1 is expressed in the Sox4+/BLBP+ progenitor population itself across development, consistent with an autocrine or paracrine mode of action.

**Figure 8:**
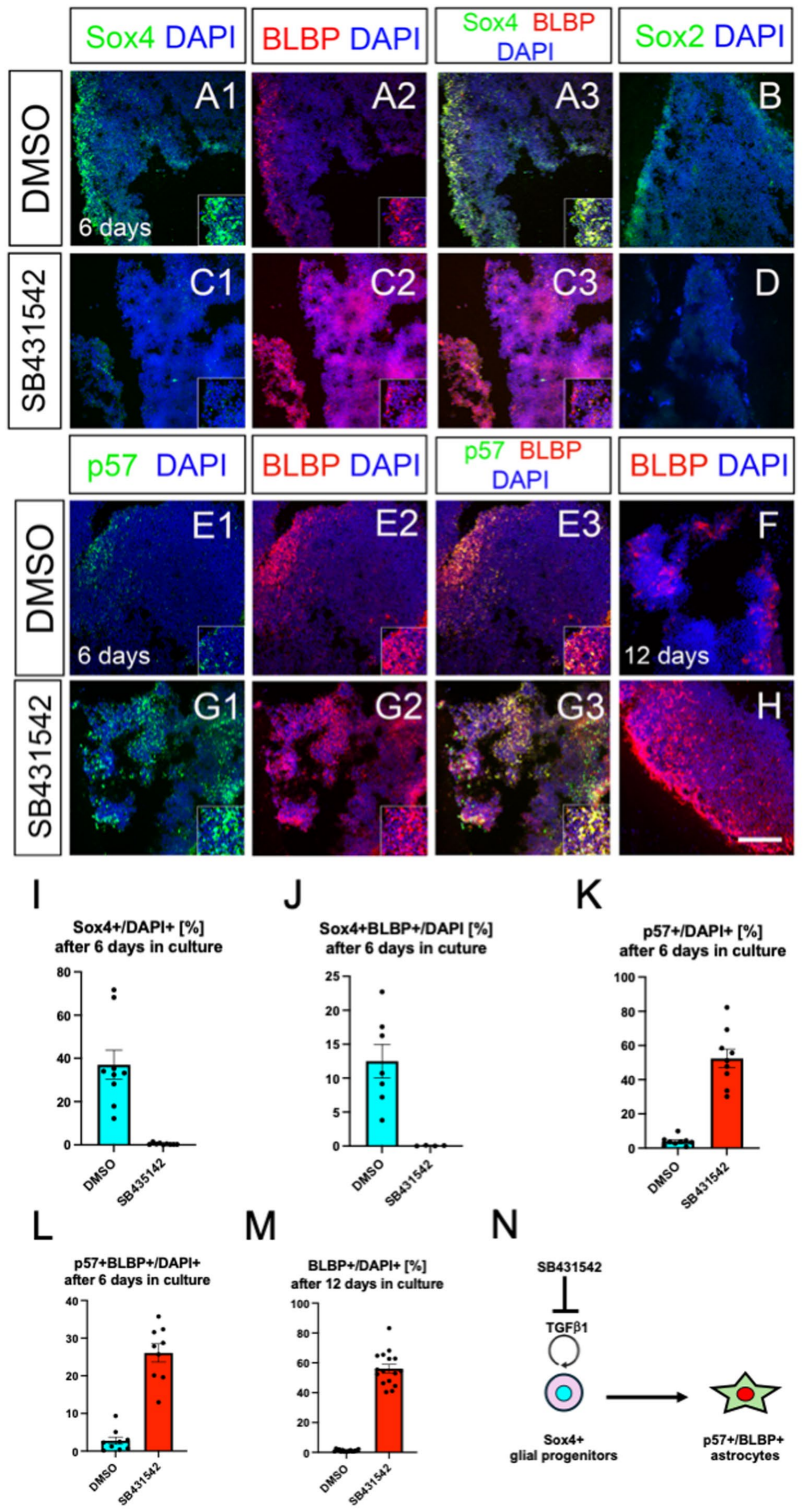
TGFβ1 signalling maintains Sox4+ progenitors and restricts the early differentiation of BLBP+ astrocytes in DG explants. P0 DG explants were treated with DMSO (control) or 10 μM SB431542 (TGFβ type I receptor inhibitor) and cultured for 6 or 12 days. **(A1–A3)** Representative images of DMSO-treated explants showing Sox4 (green), BLBP (red) and DAPI (blue) staining after 6 days. **(B)** Sox2 (green) and DAPI (blue) staining of DMSO-treated explants. **(C1–C3)** The same staining as **A1–A3** in SB431542-treated explants. **(D)** The same staining as B in SB431542-treated explants. **(E1–E3)** p57 (green), BLBP (red) and DAPI (blue) staining of DMSO-treated explants after 6 days. **(F)** BLBP (red) and DAPI (blue) staining of DMSO-treated explants after 12 days. **(G1–G3)** The same staining as E1-E3 in SB431542-treated explants. **(H)** The same staining as F in SB431542-treated explants. Insets show higher magnification of boxed areas. **(I–M)** Quantification of staining: **(I)** Sox4+ cells as a percentage of DAPI+ cells after 6 days (*n* = 6 explants, 9 sections per condition). **(J)** Sox4+/BLBP+ cells as a percentage of DAPI+ cells after 6 days (DMSO: *n* = 4 explants, 7 sections; SB431542: *n* = 4 explants, 4 sections). **(K)** p57+ cells as a percentage of DAPI+ cells after 6 days (DMSO: *n* = 4 explants, 7 sections; SB431542: *n* = 4 explants, 4 sections). **(L)** p57+/BLBP+ cells as a percentage of DAPI+ cells after 6 days (*n* = 6 explants; 9 sections per condition). **(M)** BLBP+ cells as a percentage of DAPI+ cells after 12 days (*n* = 6 explants; 16 sections per condition). Statistical comparisons by two-tailed Student’s *t*-test; exact *p* values are reported in the Results. Data are shown as mean ± SE. **(N)** Schematic model summarising the proposed mechanism: TGFβ1 produced by BLBP+ progenitors acts in an autocrine and/or paracrine manner to maintain the Sox4+ progenitor pool, thereby restricting their early differentiation into p57+/BLBP+ astrocytes. Scale bar: 200 μm in **H**.

To test whether TGFβ signalling functionally required for the maintenance of Sox4 + progenitors, we treated DG explants with SB431542, a selective inhibitor of TGFβ type I receptors for 6 days in culture. SB431542 treatment nearly eliminated Sox4+ cells in the explants (37.0% ± 6.7% of DAPI+ cells in DMSO controls versus 0.4 ± 0.1% in SB431542-treated explants; *p* < 0.0001; *n* = 6, 9 sections per condition; Figures 8A1–A3,C1–C3,I). Sox4+/BLBP+ double-positive cells were similarly depleted (12.4% ± 2.4% versus 0.05% ± 0.03%; Figure 8J). Consistent with a broader loss of the progenitor pool, Sox2+ cells were also reduced by SB431542 treatment (Figures 8B,D).

Conversely, SB431542 treatment markedly increased the p57+ early-differentiating population. p57+ cells rose from 3.9% ± 0.8% in DMSO controls to 52.4% ± 5.5% in SB431542-treated explants (*p* = 0.0025; Figures 8E1–E3,G1–G3,K), and p57+/BLBP+ cells increased from 2.7% ± 0.9% to 26.0% ± 2.4% (*p* < 0.0001; Figure 8L). Extended culture for 12 days further revealed that BLBP+ cells expanded dramatically after TGFβ inhibition (1.1% ± 0.1% in DMSO versus 56.0% ± 2.8% in SB431542; *p* < 0.0001; Figures 8F,H,M), consistent with a sustained shift from the Sox4+ progenitor state to the BLBP+ differentiating state.

Together, these data demonstrate that TGFβ signalling maintain the Sox4+ progenitor pool in the postnatal DG and thereby restricts the early differentiation of p57+/BLBP+ astrocytes (Figure 8N). The presence of TGFβ1 in the Sox4+ BLBP+ progenitors themselves suggests that this signal acts, at least in part, in an autocrine manner.

## Discussion

In this study, we identified three key features of BLBP+ astrocytic lineage development in the mouse DG. First, BLBP+ cells undergo a temporal switch from Ki67+ proliferative progenitors at P1 to p57+ early-differentiating astrocytes at P3–P6, defining a discrete window during which the proliferation-to-differentiation transition occurs. Second, the transcription factor Sox4 selectively marks BLBP+ progenitors throughout the astrocytic lineage – from bRG in the embryo, through Olig2+/BLBP+ ASPs in the early postnatal period, to RGLs at the SGZ in the adult – but is excluded from p57+/BLBP+ differentiating astrocytes and from NeuroD+ neuronal progenitors. Third, TGFβ1 is co-expressed with BLBP in these Sox4+ progenitors, and pharmacological blockade of TGFβ signalling depletes Sox4+ progenitors and accelerates their differentiation into p57+/BLBP+ astrocytes. Together, these findings establish TGFβ1-Sox4 pathway as a regulator of progenitor maintenance and differentiation timing in the BLBP+ astrocytic lineage of the DG. Whether enhanced TGFβ signaling can sustain Sox4+ glial progenitors through physical interaction between pSmad3 and the Sox4 promoter remains to be tested in future studies.

The TGFβ–Sox4 pathway was originally identified in glioma-initiating cells, in which autocrine TGFβ signalling sustains tumorigenic Sox4 expression (Ikushima et al., 2009). Our findings extend this paradigm to normal hippocampal development, demonstrating that the same pathway operates in BLBP+ progenitors of the developing DG and controls the onset of astrocyte differentiation. This parallel between glioma stem cells and normal astrocytic progenitors is consistent with the broader view that tumour-initiating cells redeploy regulatory programmes of normal stem and progenitor cells (Dongre and Weinberg, 2019). Targeted manipulation of TGFβ-Sox4 pathway in BLBP+ DG progenitors *in vivo* may therefore provide a tractable system in which to dissect mechanisms shared between normal gliogenesis and glioma initiation.

The TGFβ-Sox4 pathway appears to maintain BLBP+ progenitors through several non-mutually exclusive mechanisms. The expression of TGFβ1 in the same BLBP+ cells that express Sox4 is consistent with an autocrine action, in line with the autocrine TGFβ–Sox4 loop characterised in glioma-initiating cells (Ikushima et al., 2009), although a paracrine contribution from neighbouring cells cannot be excluded. Sox4 itself acts as a master regulator of EMT through Ezh2-mediated epigenetic reprogramming (Tiwari et al., 2013), and given that BLBP+ progenitors of the DG are highly proliferative and migratory, an EMT-like programme downstream of Sox4 may contribute to their progenitor properties. Direct testing of EMT markers such as ZO1, E-cadherin and N-cadherin in the BLBP+ lineage will be informative.

The TGFβ-Sox4 pathway is also likely to interact with Notch signalling in this context. Notch maintains the self-renewal of neural progenitors and limits their differentiation (Place et al., 2022), and pSmad3, the canonical effector of TGFβ signalling, physically interacts with the Notch intracellular domain to co-activate Notch target genes (Blokzijl et al., 2003). The expression of pSmad3 in BLBP+/pVim+ bRG of the embryonic DG (Figure 3) is consistent with a TGFβ-pSmad3 interaction with Notch signalling in BLBP+ progenitors, although the precise wiring of this network remains to be tested.

Beyond progenitor maintenance, Sox4 may also influence lineage choice within the gliogenic compartment. SOX4 inhibits oligodendrocyte differentiation of NSCs by inducing Hes5 expression (Braccioli et al., 2018), and Notch activation similarly biases progenitors towards the astrocytic over the oligodendrocyte fate. It is therefore possible that Sox4 not only maintains BLBP+ progenitors but also biases them towards the astrocytic lineage by suppressing oligodendrocyte commitment. Whether Sox4 also regulates the migratory behaviour of BLBP+ progenitors, as it does in carcinoma cells through EMT induction (Ruan et al., 2017), remains to be determined.

The BLBP+ astrocytic lineage characterised in this study is heterogeneous and only partially overlaps with the GFAP+ astrocyte population. We previously reported that pSmad3 is expressed in a subpopulation of *gfap*-GFP+ bRG, RGLs and astrocytes in the developing DG (Ohyama et al., 2023), and the present data show that pSmad3 is similarly expressed in BLBP+ bRG and ASPs. However, BLBP+/Olig2+ ASPs are largely GFAP-negative (Omura et al., 2024), and the majority of Olig2+ astrocytes in the adult mouse CNS also lack GFAP expression (Tatsumi et al., 2018; Omura et al., 2024). The BLBP+ astrocytic lineage and the GFAP+ astrocytic lineage are therefore not coextensive, and the BLBP+ progenitors identified here represent at least a partially distinct compartment.

This view is consistent with the growing recognition that astrocytes constitute a heterogeneous population defined by overlapping but non-identical marker combinations and developmental origins (Tatsumi et al., 2018; Herrero-Navarro et al., 2021; Tabata et al., 2022; Ollivier et al., 2024). Whether BLBP+ progenitors give rise predominantly to BLBP+/GFAP- astrocytes, or whether a subset transitions to a GFAP+ state during maturation, remains to be clarified. The transient co-expression of BLBP and GFAP observed in early postnatal astrocytes, together with the marked decline of BLBP expression in mature astrocytes from P14 onwards (Omura et al., 2024), suggests that BLBP may mark a developmental state rather than a stable astrocyte subtype. Lineage tracing of Sox4+ or BLBP+ progenitors will be needed to test this possibility and to define the relationship between BLBP+ and GFAP+ astrocytes in the DG.

The accumulation of p57+/BLBP+ cells at P3-P6 marks the onset of differentiation in the BLBP+ astrocytic lineage. P57 is well established as a regulator of cell cycle exit (Mairet-Coello et al., 2012) and as an inhibitor of astrocytoma growth (Tsugu et al., 2000), and its expression in BLBP+ cells at P3-P6 is therefore consistent with a transition from proliferation to a post-mitotic, differentiating state. Beyond its role in cell cycle exit, p57 also acts as a transcriptional repressor of the neurogenic gene Mash1/Ascl1 in neural progenitors (Joseph et al., 2009), which may further reinforce the astrocytic over the neuronal fate in the BLBP+ lineage.

The relationship between p57 and astrocyte subtype specification is more complex. Knockdown of p57 in adult neural stem cells reduces GFAP+ astrocyte production and instead promotes oligodendrocyte differentiation, in part through induction of the BMP antagonist Chordin (Jadasz et al., 2012), and BMP7 promotes GFAP+ astrocyte differentiation (Mabie et al., 1997). These observations suggest that BMP signalling favours the GFAP+ astrocyte fate, whereas our data show that TGFβ signalling maintains the Sox4+/ BLBP+ progenitor pool. The two pathways may therefore act antagonistically to balance progenitor maintenance against GFAP+ astrocyte differentiation, although the regulation of BLBP+/GFAP− astrocytes within this framework remains to be defined. The transient co-expression of BLBP and GFAP in some early postnatal astrocytes, and the decline of BLBP expression after P14 (Omura et al., 2024), suggests that the TGFβ-BMP balance may also influence the maturation state of astrocytes rather than only their lineage commitment. Other signals such as Shh, which upregulates p57 to promote cell cycle exit (Shkumatava and Neumann, 2005; Fu et al., 2017), may also contribute to this regulation.

The BLBP+ astrocytic lineage we have characterised also expands the emerging picture of astrocyte heterogeneity. Although classical studies treated GFAP+ cells as representative of the entire astrocyte population, recent work has identified astrocyte subtypes defined by Olig2 (Tatsumi et al., 2018), Crym (Ollivier et al., 2024) and other markers, each with distinct developmental origins and functional properties (Herrero-Navarro et al., 2021). The BLBP+/Sox4+ progenitor pool we describe here, together with its Olig2+ ASP intermediate (Omura et al., 2024), adds another layer to this picture and raises the question of whether different astrocyte subtypes in the DG arise from distinct progenitor populations or from a common pool that diverges during differentiation. Conditional manipulation of Sox4 or TGFβ1 in BLBP+ progenitors, combined with lineage tracing, will be needed to address this question.

In summary, this study identifies a temporal switch from proliferation to early differentiation in the BLBP+ astrocytic lineage of the mouse DG, and demonstrates that TGFβ signalling acts through Sox4 to maintain BLBP+ progenitors and restrict the early differentiation of astrocytes. These findings provide a framework for understanding how astrocytic progenitors are deployed during postnatal hippocampal development and provide a basis for further studies of the broader mechanisms that govern astrocyte diversity.

## Data Availability

The original contributions presented in the study are included in the article/supplementary material, further inquiries can be directed to the corresponding author.
